# Investigation of the presence of cervical human papillomavirus and the distribution of preinvasive lesions in patients using immunomodulators*-Inosine Pranobex*

**DOI:** 10.1186/s13027-026-00742-x

**Published:** 2026-02-19

**Authors:** Belma Gözde Özdemir, Gunay Safarova, Osman Yıldırım, Mercan Aslan, Fazıl Avcı, Ahmet Bilgi, Çetin Çelik

**Affiliations:** Gynecology and Obstetrics Department, Faculty of Medicine, Selçuk University, Konya, Türkiye

**Keywords:** Immunomodulator, Cervical precancerous, Malignancy, HPV, Inosine Pranobex

## Abstract

**Background:**

Human papillomavirus treatment is known to benefit from humoral and cellular immunity. It has been demonstrated that the immunomodulatory drug Inosine Pranobex (IP) increases T-cell proliferation, the number and function of natural killer cells, and the levels of T helper 1-type cytokines, including IL-2 and IFN-γ. The objective was to assess the efficacy of Inosine Pranobex in clearing HPV and precancerous lesions, as well as its effectiveness in regressing precancerous lesions.

**Methods:**

A total of 624 patients with HPV positivity and/or cervical precancerous lesions who presented to the Department of Obstetrics and Gynecology at Selçuk University Faculty of Medicine between 2022 and 2025 were evaluated. Two groups were formed: patients using it and those not using it. For patients enrolled in a given year, the results obtained in the subsequent year were considered the one-year follow-up outcomes. One-year follow-up results were analyzed.

**Results:**

HPV clearance rate was significantly higher in the Inosine Pranobex group compared to the control group (68.1% vs. 39.6%, *p* = 0.001). Viral clearance rates were significantly higher in the IP group, particularly for high-risk human papillomavirus (HPV) types, including HPV 16, HPV 18, and HPV 45. Similarly, IP treatment significantly increased HPV clearance in smokers. Regression rates of cervical lesions were higher in the Inosine Pranobex group, particularly in low-grade lesions, although the difference was not statistically significant.

**Conclusions:**

Inosine Pranobex reduces viral load and contributes to the regression of cervical lesions thanks to its antiviral and immunomodulatory effects in HPV infections. In clinical studies, a significant increase in viral clearance rates was observed during treatment. These findings indicate that the drug can be used as a complementary treatment in HPV-related pathologies.

**Clinical trial number:**

Not applicable.

## Introduction

Oncogenic types of Human Papillomavirus are known to be a significant factor in cervical cancer. Cervical precursor lesions resulting from chronic, persistent HPV infection can progress to invasive cancer if undetected and untreated [1]. Therefore, HPV must be eliminated from the body, for which competent humoral and cellular immunity is crucial. None of the women included in this study had received HPV vaccination before enrollment.

Innate immunity provides the first line of defense through the production of interferons by infected cells and the activation of dendritic cells; however, HPV’s low inflammatory profile and suppression of MHC-I expression weaken this response. Therefore, the adaptive immune response, specifically the reaction of cytotoxic CD8 + T cells against the E6 and E7 oncoproteins, plays a critical role in eliminating the infection. Cervical precursor lesions resulting from chronic, persistent HPV infection due to oncogenic types can progress to invasive cancer if undetected and untreated. In this context, immunological approaches, such as therapeutic vaccines and immune checkpoint inhibitors, aim to reactivate the immune system in the fight against HPV and represent promising strategies, particularly in the treatment of advanced lesions [2–4].

Inosine Pranobex (IP), which is a synthetic nucleoside analogue able to enhance in vitro the cell-mediated immune response, has been widely used as an immunomodulatory agent [5]. The primary drug substance used in this study is Inosine Pranobex, which enhances the cell-mediated immune response by increasing T lymphocyte activity, stimulating the production of interleukin-2 (IL-2) and gamma interferon (IFN-γ), and increasing the number and activity of cytotoxic T cells and natural killer (NK) cells. Its antiviral effects are mediated through the inhibition of viral RNA and DNA synthesis via T cell–dependent mechanisms, suppression of viral protein synthesis through ribosomal regulation, induction of apoptosis in infected cells, and promotion of lymphocyte proliferation and function, thereby facilitating a more efficient immune response to viral infections [6–8].

The study was designed to evaluate the presence of cervical HPV infection and the distribution of preinvasive cervical lesions among individuals treated with the immunomodulator Inosine Pranobex.

## Materials and methods

Patients aged 30–60 years who applied to the Department of Obstetrics and Gynecology at Selçuk University Faculty of Medicine between 2022 and 2025 were included in the study. This was a retrospective study analyzing the results of routinely applied drug treatments in the clinic. A total of 624 patients were divided into two groups: a group treated with IP and a control group not treated with IP. Among the participants, 56.7% were in the Inosine Pranobex group (*n* = 354), and 43.3% were in the control group (*n* = 270).

Patients were classified according to Inosine Pranobex use, HPV types (HPV 16, HPV 18, HPV 45, and other types), histopathological findings (LSIL and HSIL), age, parity, comorbidities, previous surgery, contraceptive methods, marital status, smoking status, age at first sexual intercourse, occupation, and comorbidities (diabetes, thyroid disorders, and hypertension). Smoking is a known risk factor for persistent HPV infection and immunosuppression. The efficacy of treatment with Inosine Pranobex was evaluated in the high-risk subgroup of smokers. Cervical smear results referred specifically to the Papanicolaou (Pap) test. Control HPV, smear, and pathology tests were taken after 1 year.

HPV testing was performed using polymerase chain reaction (PCR) targeting the L1 gene region, with an amplicon size ranging from approximately 150 to 450 bp. HPV DNA real-time PCR kits (Vitro Brand, compatible with HybriSoft HSHS versions 2.1.0R05, Spain) were used.

The generic name VELP (*500 mg Inosine Pranobex oral tablet*,* World Medicine Ilac San. ve Tic. A.Ş.*,* Istanbul*,* Turkiye*,* approved by the Ministry of Health of the Republic of Turkiye*) was administered according to the recommended dose (one tablet three times daily, or two tablets twice daily for individuals over 70 kg).

### Exclusion criteria

Patients were excluded from the study if they were taking other immunomodulators, had a history of immune deficiency, or were cancer patients.

Each patient participating in the study gave informed consent, and the study received ethical approval (07.05.2025-E.1000071).

### Statistics

Data were analyzed using IBM SPSS Statistics v22.0. Descriptive statistics are presented as frequency and percentage distributions. The chi-square (χ²) test was used to compare the distributions of HPV clearance and types between the two groups (VELP vs. Control). Fisher’s exact test was used when the expected numbers in the cells were < 5.

The relationship between smoking and HPV clearance was assessed separately for both the VELP and control groups. Cramer’s V coefficient was also calculated to determine the effect size, which was interpreted as follows (Cohen, 1988): 0.10 (small effect), 0.30 (medium effect), and 0.50 (large effect). Significance was set at *p* < 0.05 for all statistical tests.

Since the assumptions of the chi-square test were not met, when more than 20% of the expected cell frequencies were below 5, Fisher’s Exact Test was used for comparisons.

## Results

When the total characteristics were examined, the mean age of the participants was 43.0 years, with the majority falling between 30 and 40 years old (49.7%). The majority were married (77.6%). Among the participants, 32.2% had no previous births, while among those who had given birth, the most common group was women with two deliveries (28.5%). The majority of the participants had their first sexual intercourse between the ages of 18 and 19 (57.7%). A significant portion of participants (43.0%) were not using any form of contraception. Among those who used a method, the most frequently preferred method was the condom (14.0%). The group with the highest level of education was comprised of primary school graduates (40.7%), while the majority of participants (62.7%) reported an income of less than 10,000 TL. The smoking rate was 46.3% (*n* = 289), whereas 53.7% (*n* = 335) of the participants were non-smokers. While 72.1% of the participants had no additional diseases, 24.7% had them, and 3.2% had an unknown history; the history of previous surgery was absent in 75.3% of the participants, present in 19.9%, and unknown in 4.8%, as shown in Table 1. There was no significant difference in demographic characteristics between the groups.

**Table 1:** Demographic and clinical characteristics of the participants (*N* = 624)

Variable	Categories	*n*	%
Age (years)	30–40	310	49.7
	41–50	160	25.6
	51–60	154	24.7
Parity	None	201	32.2
	1 birth	60	9.6
	2 births	178	28.5
	≥ 3 births	185	29.7
Smoking status	Smoker	289	46.3
	Non-smoker	335	53.7
Age at first sexual intercourse	18–19	360	57.7
	≥ 20	264	42.3
Contraceptive method	None	268	43.0
	Condom	87	14.0
	IUD	53	8.5
	Other / Unknown	216	34.5
Education level	Primary school	254	40.7
	≥High school	370	59.3
Marital status	Married	484	77.6
	Other	140	22.4
Comorbidity	None	450	72.1
	Present	154	24.7
	Unknown	20	3.2
Previous surgery	None	470	75.3
	Present	124	19.9
	Unknown	30	4.8
Income level	< 10,000 TL	391	62.7
	≥ 10,000 TL	233	37.3

IUD, intrauterine device; BTL, bilateral tubal ligation; OCS, oral contraceptive use. Previous surgery refers to previous cesarean section

When the findings in the table are evaluated together with the numbers and percentages, the Inosine Pranobex (IP) group showed significantly higher clearance rates compared to the control group for all HPV types. For HPV16, 42 of 66 cases (63.6%) in the IP group became negative, while only 15 of 44 cases (34.1%) in the control group became negative (*p* = 0.03, effect size: medium). The difference is even more pronounced for HPV18; 26 of 37 cases (70.3%) in the IP group became negative, while only 5 of 26 cases (19.2%) in the control group became negative (*p* = 0.01, effect size: medium to high). For HPV45, 18 of 24 cases (75.0%) in the IP group became negative, while 7 of 21 cases (33.3%) in the control group became negative (*p* = 0.02, effect size: medium). In other HPV types, 155 of 227 cases (68.3%) in the IP group became negative, while 80 of 179 cases (44.7%) in the control group became negative (*p* = 0.04, effect size: medium). When all HPV types were evaluated together, clearance was detected in 241 of 354 cases (68.1%) in the IP groups. In comparison, only 107 of 270 cases (39.6%) in the control group were negative, and this difference was highly significant (*p* = 0.001, effect size: medium), as shown in Table 2.

**Table 2:** Comparison of HPV distribution and clearance rates between inosine Pranobex and control groups

HPV Type	IP Group (*n*)	Cleared *n* (%)	Control Group (*n*)	Cleared *n* (%)	*p*-value (Fisher)	Effect size
HPV16	66	42 (63.6)	44	15 (34.1)	0.03	0.25
HPV18	37	26 (70.3)	26	5 (19.2)	0.01	0.35
HPV45	24	18 (75.0)	21	7 (33.3)	0.02	0.30
Other types	227	155 (68.3)	179	80 (44.7)	0.04	0.22
**Total**	354	241 (68.1)	270	107 (39.6)	**0.001**	0.28

IP, inosine pranobex. Fisher’s exact test used when expected cell counts < 5. *p* < 0.05 considered statistically significant

IP, inosine pranobex. HPV16, HPV18, and HPV45 were analyzed individually. All HPV genotypes other than HPV16, HPV18, and HPV45 were collectively reported as other HPV types. Fisher’s exact test was used when expected cell counts were < 5. A p-value < 0.05 was considered statistically significant

The chi-square test demonstrated a statistically significant difference in HPV clearance rates between the Inosine Pranobex (IP) and control groups across all genotypes (Pearson’s χ² = 30.889, *p* < 0.05). Because more than 20% of the expected cell counts were below 5, Fisher’s exact test was considered more appropriate, confirming the significance with a p-value of 0.001. The effect size, calculated using Cramer’s V (≈ 0.28), indicated a moderate association between treatment with IP and HPV clearance.

In HPV-positive smokers (*n* = 289), clearance rates showed significant differences between the Inosine Pranobex (IP) and control groups for some genotypes. For HPV16, 28 of 32 cases (87.5%) in the IP group became negative, while only 3 of 10 cases (30.0%) in the control group became negative, and this difference was statistically significant (χ² *p* = 0.0003; Fisher *p* = 0.0011). For HPV18, 12 of 25 cases (48.0%) in the IP group became negative, whereas only 1 of 9 cases (11.1%) in the control group became negative; however, this difference did not reach statistical significance (χ², *p* = 0.0509; Fisher’s *p* = 0.107). For HPV45, 11 of 12 cases (91.7%) in the IP group became negative, while only 2 of 6 cases (33.3%) in the control group became negative, and this difference was found to be significant (χ² *p* = 0.0092; Fisher *p* = 0.0217). For other HPV types, 91 of 109 cases (83.5%) in the IP group became negative, while only 22 of 86 cases (25.6%) in the control group became negative, and the difference was highly significant (χ² *p* < 0.001; Fisher *p* < 0.001), as shown in Table 3.

**Table 3:** HPV clearance rates according to HPV type in smokers with HPV positivity (*n* = 289)

HPV Type	IP Group Cleared *n*/*N* (%)	Control Group Cleared *n*/*N* (%)	χ² *p*-value	Fisher *p*-value
HPV16	28/32 (87.5)	3/10 (30.0)	0.0003	0.0011
HPV18	12/25 (48.0)	1/9 (11.1)	0.0509	0.1070
HPV45	11/12 (91.7)	2/6 (33.3)	0.0092	0.0217
Other HPV types	91/109 (83.5)	22/86 (25.6)	< 0.001	< 0.001
Total	142/178 (79.8)	28/111 (25.2)	—	—

IP, inosine pranobex. Statistical significance defined as *p* < 0.05

IP, inosine pranobex. HPV16, HPV18, and HPV45 were analyzed individually. All HPV genotypes other than HPV16, HPV18, and HPV45 were reported collectively as other HPV types. Statistical significance was defined as *p* < 0.05

In the analysis of ordinal variables, the Gamma coefficient indicated a weak negative association that was not statistically significant (Γ = −0.12, *p* = 0.923). This result demonstrates that there was no significant monotonic relationship between HPV genotype categories (HPV16, HPV18, HPV45, and other types) and smoking status in the study population. Similarly, Somers’ coefficient, with cigarette use as the dependent variable, was also non-significant (d = -0.08, *p* = 0.923), indicating the absence of a systematic linear relationship between HPV genotype distribution and smoking behavior.

When low-grade squamous intraepithelial lesion (LSIL) cases were evaluated histopathologically, persistent LSIL was observed in 12.8% of patients in the Inosine Pranobex (IP) group and in 27.3% of patients in the control group. LSIL regression occurred in 87.2% of cases in the IP group and in 72.7% of cases in the control group. However, this difference did not reach statistical significance (*p* > 0.05); therefore, no conclusive inference can be made regarding the effect of treatment on LSIL regression. In the high-grade squamous intraepithelial lesion (HSIL) group, persistent disease was detected in 15 of 67 cases (22.4%) in the IP group, with regression observed in 52 cases (77.6%). In the control group, persistent HSIL was present in 7 of 33 cases (21.2%), while regression was observed in 26 cases (78.8%). No statistically significant difference was observed between the groups (*p* = 1.000), as shown in Table 4.

**Table 4:** Histopathological outcomes of LSIL and HSIL cases according to treatment group

Lesion Type	Outcome	Inosine Pranobex (IP) *n* (%)	Control *n* (%)	*p*-value
LSIL	Persistent	**6/47 (12.8%)**	**6/22 (27.3%)**	> 0.05
	Regression	**41/47 (87.2%)**	**16/22 (72.7%)**	
HSIL	Persistent	15/67 (22.4%)	7/33 (21.2%)	1.000
	Regression	52/67 (77.6%)	26/33 (78.8%)	
Total	—	114 (LSIL+HSIL)	55 (LSIL+HSIL)	—

Values are presented as number and percentage. Total indicates the total number of LSIL and HSIL cases evaluated in each group. No statistically significant differences were observed between the groups

No serious adverse effects related to Inosine Pranobex were reported by patients during the treatment period.

## Discussion

The results of the present study demonstrated that Inosine Pranobex is associated with increased HPV clearance, particularly in high-risk genotypes and among smokers. A detailed review of the literature’s findings clearly demonstrates the need for immunomodulators in the clearance of HPV. One study has indicated that immunomodulators strengthen the immune response by reducing viral load, particularly in cases of chronic HPV [9]. Our findings align with these results, suggesting that Inosine Pranobex, through its immunostimulating effects, contributes to viral clearance and may play a protective role against HPV’s immune evasion mechanisms. The results obtained indicate that Inosine Pranobex increases HPV clearance and is particularly effective in high-risk HPV types. Similarly, the literature reports that Inosine Pranobex use provides both clinical and virological improvement in HPV-related lesions, with a decrease in viral load during treatment [10]. Another study has shown that the drug’s antiviral and immunomodulatory properties contribute to HPV elimination by supporting cellular immune responses. Our study findings regarding HPV clearance are consistent with these results. Suggesting that Inosine Pranobex may be particularly effective in eliminating high-risk HPV types and may help reduce infection by strengthening the immune response [10, 11].

Inosine Pranobex enhances cell-mediated immune responses, including T lymphocyte activity and Th1-type cytokine production, which may contribute to improved HPV elimination. At the molecular level, IP has been reported to suppress viral RNA and DNA synthesis, inhibit viral protein synthesis at the ribosomal level, and induce apoptosis in infected cells [12]. These multifaceted effects partially offset HPV’s immune evasion mechanisms (such as reduced antigen expression and suppression of the interferon response), facilitating viral clearance. The significant increase in clearance observed in high-risk HPV types and smokers in this study also seems compatible with these immunological mechanisms [13]. HPV chronicity and progression to cervical intraepithelial lesions are associated with immune system evasion mechanisms of viral oncoproteins. E6 and E7 oncoproteins trigger cellular oncogenic processes, making it difficult for the immune system to recognize and eliminate viral cells. Similarly, it has been emphasized that HPV weakens the immune response by suppressing the interferon response and low antigen expression [14]. The increased clearance achieved by Inosine Pranobex in high-risk HPV types suggests that it partially counterbalances these immune evasion mechanisms and contributes to infection regression by enhancing the Th1-biased cytokine response [15, 16].

In high-grade lesions, HPV’s immune system evasion strategies and genetic/epigenetic changes become dominant [17, 18]. It has been emphasized that in the advanced stages of cervical intraepithelial neoplasia, the immune response becomes weakened, and immune cells become insufficiently effective in the infected tissue. It has been reported that carcinogenic HPV types become chronic, hiding from the immune system through low antigen expression and suppressed interferon response [19]. E6/E7 oncoproteins have been shown to deactivate tumor suppressor mechanisms, such as p53 and pRb, thereby disrupting the cellular cycle and leading to both cell immortalization and escape from immune recognition [20]. In this context, while Inosine Pranobex ‘s enhancement of the Th1 response and activation of cytotoxic T cells and NK cells may contribute to viral clearance in early and mid-stage infections, the multilayered immune evasion mechanisms of the virus in advanced precancerous lesions, such as HSIL, limit the effectiveness of treatment. In other words, the problem is not so much the inadequacy of the drug itself, but rather the biological escape of advanced lesions from immune control. Therefore, the failure of IP to achieve the expected level of efficacy in HSIL cases can be attributed to the mechanisms described in the literature, including suppression of viral oncoproteins, immune evasion strategies, and advanced transformation processes.

In the study, HPV clearance rates were significantly increased compared to the control group (68.1% vs. 39.6%), particularly in high-risk HPV types (HPV16, HPV18, and HPV45) and in the smoker subgroup. A large-scale, multicenter study evaluated 5650 patients and found a 54.8% HPV clearance rate with IP monotherapy, while this rate increased to 84.2% when IP was added to routine therapy [21]. This result demonstrates that combination therapy provides greater clinical efficacy compared to monotherapy. In our study, IP was used in monotherapy, and after one year, HPV genotypes and cervical pathology were evaluated. In another study, the effectiveness of immunotherapy after conization was investigated. Inosine Pranobex administration increased clearance of high-risk HPV genotypes and reduced HSIL recurrence rates from 21.9% to 12.5% ​​over a four-year follow-up period [22]. These data highlight the role of immunomodulators as an adjuvant treatment approach, particularly in the postsurgical period. This study evaluated HPV and pathology at 1 year. The absence of long-term follow-up represents a limitation of this study. When interpreted in the context of existing literature, the findings suggest that Inosine Pranobex may be effective as monotherapy, while previous studies indicate that combination or adjuvant use could provide additional benefits. Therefore, Inosine Pranobex can be considered an effective immunomodulatory option for HPV treatment, both as a standalone treatment and in combination protocols.

Persistent HPV infection represents a critical biological process underlying the progression from initial viral exposure to cervical carcinogenesis, particularly in infections caused by high-risk genotypes [23]. In this context, therapeutic strategies that enhance host immune competence may play a supportive role by improving viral control and reducing the likelihood of chronic infection, especially in individuals predisposed to immune dysregulation [24].

The results also reveal that HPV18 responds more significantly to treatment compared to other types. HPV18’s biologically potent oncoprotein activity and immune evasion mechanisms place a more significant burden on the immune system. Therefore, enhancing the immune response may play a more critical role in HPV18 clearance. Furthermore, mechanisms such as epigenetic regulation and methylation have been reported to influence HPV gene expression and the host’s immune response, potentially leading to differences in treatment response [25]. The high clearance rates observed in HPV-18 suggest that immune modulation may have a significant impact on this genotype.

Cigarette smoking is a significant risk factor for the persistence of HPV infections. Nicotine and its byproducts have been shown to suppress the immune response, reduce interferon production, and weaken antiviral defense mechanisms. This facilitates chronic infection and promotes the progression of cervical intraepithelial lesions. Furthermore, it has been reported that smoking can reduce treatment response by affecting drug metabolism at the pharmacokinetic level [26]. Therefore, decreased viral clearance rates in HPV-positive smokers are an expected outcome.

This study demonstrates that Inosine Pranobex has a significant effect on HPV clearance, with efficacy particularly pronounced in high-risk types (HPV 16, HPV 18, HPV 45) and in the subgroup of smokers. It has been reported that Inosine Pranobex immunotherapy enhances cellular immunity by strengthening the Th1 type immune response, thereby facilitating viral clearance in cervical HPV-positive patients [27]. Similarly, recent clinical studies have shown that Inosine Pranobex immunotherapy significantly increases the clearance of high-risk HPV types after cervical conization and can reduce recurrence rates of high-grade lesions. It is considered an important treatment strategy in preventing HPV persistence. In the present study, the detection of higher HPV clearance rates in patients treated with Inosine Pranobex, especially in the subgroup of smokers with impaired immune responses and high-risk HPV genotypes, is consistent with these current findings reported in the literature [28].

While it does not provide an additional advantage in advanced-stage lesions (HSIL), the increased regression rates in low-stage lesions demonstrate the drug’s clinical value. The findings support the use of Inosine Pranobex as a complementary treatment option, either alone or in combination protocols. It is essential to note that individual differences and variable drug responses are possible, and caution should be exercised, particularly in individuals with a history of immunological disease.

## Conclusion

As an immunomodulator, Inosine Pranobex supports HPV clearance by enhancing the immune response and may contribute to individualized management strategies for HPV-related conditions. Although smoking cessation remains the most effective strategy to improve HPV clearance in smokers, Inosine Pranobex may be considered as a supportive immunomodulatory option in selected patients.

## Data Availability

The datasets generated and/or analyzed during the current study are not publicly available due to institutional restrictions, but are available from the corresponding author upon reasonable request.
